# Pembrolizumab-Induced Immune-Mediated Glossitis

**DOI:** 10.7759/cureus.21708

**Published:** 2022-01-29

**Authors:** Alwin Alias, James A Hall, Pruthali Kulkarni, Alan C Gowan

**Affiliations:** 1 Hematology and Medical Oncology, Baylor Scott & White Medical Center, Temple, USA; 2 Internal Medicine, Baylor Scott & White Medical Center, Temple, USA; 3 Medical Oncology, Baylor Scott & White Medical Center, Temple, USA

**Keywords:** corticosteroids, prednisolone, immunotherapy, chemotherapy, keytruda, immune-mediated glossitis, pembrolizumab

## Abstract

Pembrolizumab (Keytruda), an anti-PD-1 antibody used in the treatment of several different malignancies has been identified to cause adverse effects pertaining to multiple body systems which include respiratory, gastrointestinal, dermatologic, and endocrine manifestations known as immune-related adverse events (IRAEs). Skin manifestations have been most described in current literature highlighting the most common adverse effects of this agent. However, adverse outcomes involving the oral mucosa have been rarely identified in the PD-1 and PD-L1 inhibitor classes of immunotherapeutic agents. We present a case of a 71-year-old male who was treated with a chemotherapeutic regimen including pembrolizumab for newly diagnosed squamous cell carcinoma of the lung, who later developed ulcerations on his tongue that were consistent with glossitis. Upon determining that this adverse effect may be immune-related, the patient was treated with oral prednisone 40 mg with a 10 mg taper each subsequent week, which resulted in significant improvement in the patient’s symptoms following one month of treatment.

## Introduction

Pembrolizumab, also known as Keytruda, is a humanized monoclonal anti-PD-1 antibody that has been widely used in the treatment of numerous malignancies including melanoma, non-small cell lung cancer, and a variety of other solid tumors and lymphomas. Programmed cell death protein 1 (PD-1) is a checkpoint protein found on T cells that normally acts as a modulator in preventing the body’s native T cells from attacking other native cells and tissues [1]. When PD-1 is ligated by PD-L1 and PD-L2 found on neoplastic cells, a gateway is opened for tumor cells to evade the body’s defenses against them, allowing unchecked proliferation of tumor cells [1]. Pembrolizumab acts by blocking the activity of PD-1, and preventing tumor cells from evading the body’s innate defenses against tumor cells. Immune-related adverse effects have been widely described and identified with the use of PD-1 inhibitors like pembrolizumab and PD-L1 inhibitors like nivolumab, which includes (but is not limited to) thyroid dysfunction, hepatitis, pneumonitis, and paresthesia [1]. The most common of these adverse effects, however, were dermatological manifestations which included pruritis, alopecia, and lichen planus [1]. Oral mucosal changes have been rarely reported with xerostomia, lichenoid lesions, and dysgeusia being most seen. Less than 0.1% of cases reported glossitis as an adverse effect of pembrolizumab [2].

The content of this case report was previously presented as a poster at the Baylor Scott & White Scholar’s event on May 7, 2021.

## Case presentation

**Figure 1:**
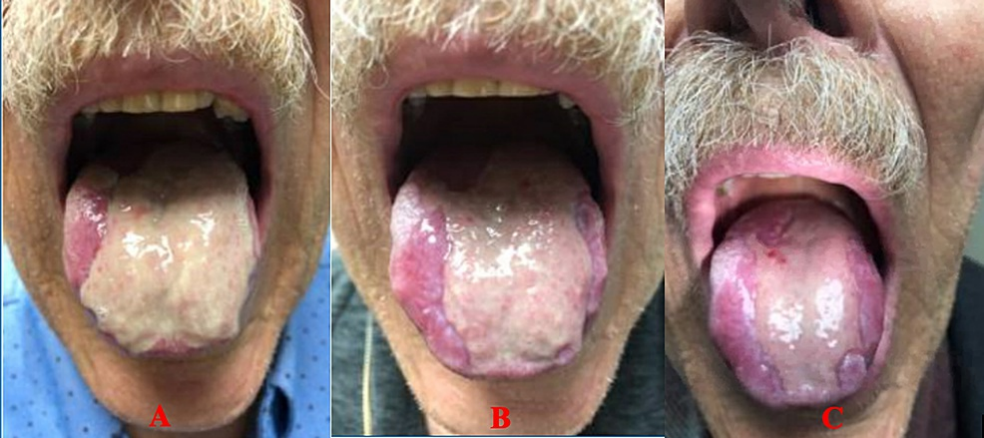
Pembrolizumab-induced glossitis -- treatment progression with oral corticosteroids. Pane A depicts the baseline of the patient’s symptoms prior to starting treatment with oral corticosteroids. Pane B depicts improvement in his symptoms following 14 days of corticosteroid treatment. Pane C depicts near-complete resolution of the patient’s symptoms following one month of corticosteroid treatment.

We present the case of a 71-year-old Caucasian male with a medical history of stage III (T3N1M0) squamous cell carcinoma of the larynx, coronary artery disease, diabetes mellitus, hypertension, hyperlipidemia, hypothyroidism, and peripheral vascular disease, who had completed treatment for his laryngeal cancer with concurrent weekly cisplatin and radiation. Follow-up positron emission tomography (PET)/CT after treatment revealed no evidence of metastatic or residual disease. Nine months after completing treatment of his cancer, he was hospitalized for respiratory symptoms, where workup found a pulmonary embolus and a right-sided moderate pleural effusion. Subsequent cytology of the pleural fluid revealed a non-small cell carcinoma with immunohistochemistry further delineating a squamous cell carcinoma (metastatic from previous laryngeal cancer vs. new primary lung malignancy). Following this diagnosis, he was treated with four cycles of carboplatin, paclitaxel, and pembrolizumab and subsequently planned for maintenance pembrolizumab. The most recent PET/CT revealed no evidence of active disease. Following 12 cycles of pembrolizumab maintenance therapy, he began to develop well-demarcated ulcerations on the dorsal surface of his tongue which progressively worsened and prompted immediate discontinuation of further immunotherapy with pembrolizumab after a total of 15 cycles (Figure 1). 

Considering this rather atypical adverse effect, the patient was subsequently treated with triple mix mouthwash and fluconazole, which resulted in little to no improvement in his symptoms. We reached the differential diagnoses of immune-mediated glossitis, paraneoplastic pemphigus, and oral lichen planus for this rare manifestation. Upon investigating an immune-related source to this adverse effect as a result of pembrolizumab, he was then started on prednisone 40 mg by mouth daily, tapered by 10 mg each week, which yielded progressively significant improvement in his symptoms. The emergence of this adverse effect resulted in the discontinuation of the patient's maintenance therapy to prevent worsening of the glossitis and to focus on the treatment of the glossitis itself. The CT scans following the 15 cycles of pembrolizumab maintenance therapy showed no evidence of residual disease, so the disruption in therapy did not result in any significant changes in the patient's oncological status or overall treatment at the time. 

## Discussion

This clinical vignette depicts the development of glossitis resulting from an immune-related phenomenon following treatment with the PD-1 inhibitor pembrolizumab. Although skin and dermatological manifestations have been widely reported as adverse effects in the pharmacological profiles of PD-1 and PD-L1 inhibitors, adverse outcomes involving the oral mucosa have been rare and under-reported with these classes. A single institutional retrospective cohort study found a large incidence of oral mucositis as an adverse effect with the combination of anti-estimated glomerular filtration rate (EGFR) agents such as cetuximab and panitumumab with 5-fluorouracil (5-FU) in the treatment of colorectal cancer [3]. This study made further conclusions in panitumumab being a bigger culprit than cetuximab in inducing this adverse outcome. Likewise, oral mucosal toxicities have been described with the use of tyrosine kinase inhibitors (TKIs) and vascular endothelial growth factor (VEGF) inhibitors [4]. Another review also highlighted a case of glossitis in a patient who received just one cycle of nivolumab [5]. Preventative prophylactic strategies in reducing the incidence of oral mucositis in patients receiving chemotherapy and radiation have been recently described in the literature, and some institutions have even adopted them as the standard of care in preventing this adverse effect. One prospective study analyzed the efficacy of a mouth rinse consisting of a mixture of soluble prednisolone, nystatin, and saltwater prior to starting chemotherapy with FEC (5-Fluorouracil, Epirubicin, Cyclophosphamide) for breast cancer, in reducing the incidence of oral mucositis following treatment [6]. The study found only two cases out of 68 included patients who developed clinically significant oral mucositis following prophylactic treatment with the mouthwash. A literature review of 60 clinical studies studying preventative measures for oral mucositis also found prophylactic administration of honey, glutamine, and zinc prior to chemotherapy and radiation reduced the risk of developing oral mucositis as well [7].

## Conclusions

This case highlights the use of oral steroids followed by appropriate tapering of steroids in the treatment of immune-mediated glossitis induced by pembrolizumab. This adverse effect is one that has been rarely reported with this immunotherapeutic agent, which further sheds light on the ways in which immunomodulatory drugs can induce a wide range of side effects across different patients. These drugs are typically used in the treatment of several different cancers, and these patients are already much more susceptible to experiencing more adverse outcomes from these drugs given their immune status and diffuse involvement of many of these cancers. This case also highlights the potential methods and agents that can be used as prophylaxis in preventing these immune-related adverse effects. Thinking about the patient’s overall health and outcome, it is always recommended to think ahead about how the various therapeutic modalities available can affect a patient’s future health while treating primary cancer they suffer from.
